# Combined 3D bioprinting and tissue-specific ECM system reveals the influence of brain matrix on stem cell differentiation

**DOI:** 10.3389/fcell.2023.1258993

**Published:** 2023-10-20

**Authors:** Martina Zamponi, Peter A. Mollica, Yara Khodour, Julie S. Bjerring, Robert D. Bruno, Patrick C. Sachs

**Affiliations:** ^1^ School of Medical Diagnostic and Translational Sciences, College of Health Sciences, Old Dominion University, Norfolk, VA, United States

**Keywords:** cellular microenvironment, extracellular matrix, brain, fate determination, neural differentiation, 3D bioprinting, stem cells

## Abstract

We have previously shown that human and murine breast extracellular matrix (ECM) can significantly impact cellular behavior, including stem cell fate determination. It has been established that tissue-specific extracellular matrix from the central nervous system has the capacity to support neuronal survival. However, the characterization of its influence on stem cell differentiation and its adaptation to robust 3D culture models is underdeveloped. To address these issues, we combined our 3D bioprinter with hydrogels containing porcine brain extracellular matrix (BMX) to test the influence of the extracellular matrix on stem cell differentiation. Our 3D bioprinting system generated reproducible 3D neural structures derived from mouse embryonic stem cells (mESCs). We demonstrate that the addition of BMX preferentially influences 3D bioprinted mESCs towards neural lineages compared to standard basement membrane (Geltrex/Matrigel) hydrogels alone. Furthermore, we demonstrate that we can transplant these 3D bioprinted neural cellular structures into a mouse’s cleared mammary fat pad, where they continue to grow into larger neural outgrowths. Finally, we demonstrate that direct injection of human induced pluripotent stem cells (hiPSCS) and neural stem cells (NSCs) suspended in pure BMX formed neural structures *in vivo*. Combined, these findings describe a unique system for studying brain ECM/stem cell interactions and demonstrate that BMX can direct pluripotent stem cells to differentiate down a neural cellular lineage without any additional specific differentiation stimuli.

## Introduction

The microenvironment influences various cellular processes, such as cell migration and differentiation (Chastain et al., 2006; Clause et al., 2010; Chaudhuri et al., 2016; Wang et al., 2016). The histological organization of tissues and the disposition of cells in a natural three-dimensional context affects the tissue’s biological functions and biochemical specificities (Khoruzhenko, 2011; Duval et al., 2017). A major component of the cellular environment is the extracellular matrix (ECM), a three-dimensional network of resident cell-secreted macromolecules. The ECM is a fundamental determinant of cell behavior, engages in cell signaling, and facilitates cell motility and structural organization (Vorotnikova et al., 2010; Crapo et al., 2012; Theochari et al., 2016). Furthermore, due to the complex and varied nature of the types and quantities of the macromolecules contained in each tissue’s ECM, they each have a unique molecular signature (Bruno et al., 2017; Mollica et al., 2019). Traditional substrates for two- and three-dimensional cell cultures use ECMs or alternate materials (e.g., rat tail collagen, Geltrex/Matrigel, Alginate, etc.) (Caliari and Burdick, 2016; Sun et al., 2016; Yan et al., 2017). These matrices present various limitations, as they lack the biomimetic specificity of tissue ECMs. Therefore, the use of tissue-specific ECMs, which allow a more faithful reproduction of the *in vivo* environment, has been a developing field of research in recent years (Sachs et al., 2017a; Mollica et al., 2019).

The cellular microenvironment has a fundamental role in establishing stem cell fate and regulating the behavior of differentiated cells. Our group and others have shown that the microenvironment is the controlling factor in cell-fate determination. For instance, the mammary microenvironment redirects non-mammary stem cells and cancer cells to adopt normal and functional mammary epithelial cell fates (Boulanger et al., 2007; Booth et al., 2008; Bussard et al., 2010; Booth et al., 2011; Boulanger et al., 2012; Boulanger et al., 2013; Bruno et al., 2017). The whole complexity of the *in vivo* microenvironment is currently impossible to fully recreate *in vitro*, consisting of paracrine and cell-to-cell signaling between the stroma, parenchyma, ECM, and other factors originating from outside the microenvironment. The ECM, however, is produced by the interactions of many of these factors and thus offers a unique milieu of factors that can represent a “snapshot” of signaling in the tissue that researchers can exploit to study stem cell differentiation. For example, the simple addition of mammary ECM to the mammary fat pad stromal environment is sufficient to differentiate testicular and ESC cells into mammary epithelial cell fates *in vivo* (Bruno et al., 2017). This remarkable finding underscores the power of the ECM in cell fate determination. Understanding these interactions can lead to better approaches to regenerative medicine, basic stem cell, aging, and cancer research.

A few groups have successfully explored the potential use of central nervous system-derived hydrogels as a substrate for neural cell cultures (DeQuach et al., 2011; Crapo et al., 2012; Medberry et al., 2013; Simsa et al., 2021), with some extensive characterization of the protein composition (Simsa et al., 2021). However, central nervous system ECM’s influence on stem cell differentiation has not been fully explored, and robust 3D culture systems that use brain specific ECMs are lacking. Such systems are required for studies on the role of the microenvironment in cellular differentiation and tissue development. An ideal system would allow for reproducible 3D structures that support neuronal differentiation and transplantability *in vivo*.

We have previously described a simple, accessible bioprinting system that can vastly improve the control and reproducibility of mammary epithelial organoid formation (Reid et al., 2016; Reid et al., 2018; Mollica et al., 2019; Reid et al., 2019). Combined with mammary-specific ECM, this system supports unique morphological and cellular changes that better mimic the *in vivo* environment (Mollica et al., 2019). Here, we combine our 3D bioprinting approach with the addition of brain-derived ECM (BMX) to produce a novel culture system. Furthermore, we take advantage of mouse embryonic stem cells (mESCs) that express green fluorescent protein from the oligodendrocyte lineage marker OLIG2 (mESC-OLIG2-GFP) (Xian et al., 2003). OLIG2 is a critical mediator of oligodendrocyte differentiation, but the gene is also expressed in early embryonic development of neural lineages (Mizuguchi et al., 2001) and is seen in NSCs (Hong et al., 2016; Kageyama et al., 2019) and motor neuron progenitors (Mizuguchi et al., 2001; Novitch et al., 2001). Thus, this system can allow real-time tracking of general differentiation toward neural lineages. Using this system, we show that adding BMX to Geltrex (GTX) hydrogels influences the differentiation of mESCs to neural cell fates but has minimal effect on NSCs. Furthermore, we show that mESCs bioprinted into BMX-positive gels form large primitive neural structures when transplanted into the cleared mouse mammary fat pad. Conversely, those printed into BMX-negative gels (GTX only) either failed to survive or formed teratomas *in vivo*. Furthermore, using injectable forms of BMX, we demonstrated that human iPSCs and NSCs are directed to form large neuroectoderm structures *in vivo*. This data describes a unique high throughput culture system for studying brain ECM/cellular interactions and demonstrates the capacity of brain ECM to help direct the differentiation of pluripotent stem cells to neuronal cell fates.

## Materials and methods

### Preparation of porcine brain ECM scaffolds

Porcine brains were extracted from adult animals obtained from three sources: local butcher (Chesapeake, VA), Animal Biotech Industries, Inc. (Danboro, PA), and sacrificed animals from the research laboratory of Dr. Christian Zemlin at the Frank Reidy Center for Bioelectrics. Brains were carefully extracted, and the meninges and the superficial blood vessels were removed. The tissue was washed in sterile water to remove residual blood. The tissue was cut into sections and washed in a 2% v/v solution of N-Lauryl Sarcosine (NLS) sodium salt solution (Sigma Aldrich) and 2X Antibiotic-Antimycotic (ABAM; Thermo Fisher). All washes were performed at room temperature in a temperature-controlled orbital shaker (MaxQ 4000; Thermo Scientific). The supernatant was decanted every 24 h and replaced with fresh decellularizing solution for 3–7 days (depending on the tissue sample size) until complete cell removal. The slurry was centrifuged at 35,000 x g for 5 min. The supernatant was carefully decanted to prevent the loss of ECM, and the product was resuspended and rinsed with ultrapure water. A total of ten washes were performed to ensure the complete removal of the NLS solvent. Subsequently, the product was washed once with isopropyl alcohol for 24–48 h to remove any loose lipid components. Finally, the product was washed ten times with ultrapure water to remove any residual alcohol. The decellularized product was lyophilized using a FreeZone Triad Freeze Dryer (Labconco). 10 mg/mL pepsin (from porcine mucosa, Sigma Aldrich) solution in 0.1M HCL was used to digest 5 mg/mL of ECM for 24–72 h. The acidic product was dialyzed using a 6–8 kDa dialysis tubing (Spectra Por; Spectrum Labs, Virginia Beach, VA) against a neutral PBS solution with 1X ABAM. Dialysis was performed at 4.0 °C to prevent premature gelling. The product obtained was self-gelling when kept at 37.0°C for 30 min. Removal of genomic DNA was verified visually by hematoxylin and eosin staining of paraffin-embedded sections and by the Quant-iT™ PicoGreen^®^ dsDNA Reagent Kit (Invitrogen). The assay was performed following the manufacturer’s instructions, and fluorescence was measured using a Varioskan™ Lux microplate reader (Thermo Fisher Scientific).

### Characterization of brain-derived ECM

The total protein concentration of the final brain ECM product was characterized using the DC™ Protein Assay (Bio-Rad Laboratories), following the manufacturer’s instructions. The product concentration was measured spectrophotometrically using a Nanodrop 2000 (Thermo Fisher) and a wavelength of 750 nm.

Qualitative characterization of the BMX hydrogels’ protein content, compared to the commercially available GTX, was done by performing a polyacrylamide gel electrophoresis (PAGE) assay using a 7.5% polyacrylamide gel (Bio-Rad Laboratories). Samples were prepared as a serial dilution of the brain matrix product, 100%–6.5% concentration of product to sterile water. GTX was diluted 1:100 in sterile water for a final 1.57 mg/mL concentration. Gels were visualized with a coomassie brilliant blue G-250 dye (Amresco, Solon, OH) per manufacturer protocol. The stained gels were imaged with a myECL™ Imager (Thermo Fisher Scientific).

Gels were analyzed for rheological properties using an Anton Paar MCR 302 rheometer. Using the parallel plate attachment the gels were subject to a frequency sweep to determine G′ and G’’. GTX gels were prepared at a 12 mg/mL concentration as recommended by the manufacturer. BMX + gels were prepared as described below.

### Generation and culture of human iPSC-derived neural stem cells

The human induced pluripotent stem cell (hiPSC) line bASC3 was previously generated in our laboratory from human breast adipose stem cells (Mollica et al., 2018a). hiPSCs were adapted to feeder-free conditions, and cells were seeded on GTX-coated plates. hiPSC cultures were maintained following the Maintenance of Human Pluripotent Stem Cells in mTeSR™1 technical manual (STEMCELL Technologies; Cambridge, MA). Cells were maintained in complete mTeSR™ Plus medium (STEM CELL Technologies), replenished daily. Enzymatic passage was performed using Dispase (STEMCELL Technologies) according to the manufacturer’s suggested protocol. Pluripotency of the hiPSC colonies was confirmed using a Tra-1-60 live staining kit (Thermo Fisher).

Human neural stem cells (hNSCs) were generated from the is-bASC3 line using the STEMdiff™ Neural System Embryoid Body (EB) protocol (STEMCELL Technologies) following the manufacturer’s instructions. Newly formed NSCs were cultured on GTX^®^ coated plates and in complete StemPro^®^ NSC Serum Free Medium (Thermo Fisher Scientific), replenished every other day. Cells were passaged using TrypLE™ Express Enzyme (Thermo Fisher) according to the manufacturer’s suggested protocol. The identity of the newly formed hNSCs was confirmed via immunocytochemistry using the neural stem cell markers SOX2 and NESTIN.

### Generation and culture of mouse ESC-derived neural stem cells

The mouse embryonic stem cell (mESC) OLIG2-GFP reporter line (mESC-OLIG2-GFP) was purchased from ATCC^®^ (SCRC-1037). mESC-OLIG2-GFP were adapted to feeder-free conditions, and cells were seeded on GTX coated plates and cultured in mESC Cell Culture medium (Mouse ES Cell Basal Medium (ATCC), 15% Embryonic Stem Cell qualified FBS (Life Technologies), 1X Antibiotic-Antimycotic (Thermo Fisher), 55 µM β-Mercaptoethanol (Gibco), 1000U/mL of Leukemia Inhibitory Factor (LIF) supplement (Millipore Sigma), 1 µM MEK inhibitor PD0325901 (Millipore Sigma) and 5 µM GSK3 inhibitor CHIR99021 (Millipore Sigma)). The cell culture medium was replenished daily. Cells were passaged using TrypLE™ Express Enzyme according to the manufacturer’s suggested protocol. The pluripotency of the mESC colonies was confirmed using an SSEA-1 live staining kit (Esi Bio).

Mouse neural stem cells (mNSCs) were generated from the G-Olig2 line using the STEMdiff™ Neural System Embryoid Body (EB) protocol and following the manufacturer’s instructions. Newly formed NSCs were cultured on GTX^®^ coated plates and in complete StemPro^®^ NSC Serum-Free Medium, replenished every other day. Cells were passaged upon reaching 80%–90% confluency, using TrypLE™ Express Enzyme according to the manufacturer’s suggested protocol. The identity of the newly formed mNSCs was confirmed via immunocytochemistry using the neural stem cell markers SOX2 and NESTIN.

### Neural differentiation

Neural differentiation was achieved following previously published protocols (Dimos et al., 2008; Ehrlich et al., 2017). Briefly, NSCs were cultured in Neurobasal™ Medium (Thermo Fisher Scientific), supplemented with 2% B-27™ Supplement (50X) (Thermo Fisher Scientific), 0.5 mM GlutaMAX™, 1X ABAM, 500 nM purmorphamine (STEMCELL Technologies), 50 nM retinoic acid (STEMCELL Technologies). Partial (1/2) medium change was performed daily. At Day 15 of differentiation, the medium was substituted with neural maturation medium, constituted of Neurobasal™ Medium supplemented with 2% B-27™ Supplement (50X), 0.5 mM GlutaMAX™, and 1X ABAM. Cells were differentiated for up to 30 days. The ability of the cells to undergo neural differentiation onto different substrates was evaluated via qRT-PCR and immunocytochemistry assays.

### 2D plating of brain ECM for cell culture

Cell culture plates or glass coverslips were coated with a 0.1% polyethyleneimine (PEI; Sigma) solution in borate buffer pH 8.4 (Deneault et al., 2018) and incubated at 37°C for 1 hour. The wells were washed thrice with sterile water for 5 minutes. Porcine-derived hydrogels were diluted to a 0.3 mg/mL concentration in cold DMEM/F12 and incubated overnight at 37°C. The wells were then washed again three times with sterile water for 5 minutes. Cells were seeded, cultured, and differentiated following the previously described protocols.

### Three-dimensional cell culture in brain ECM-derived substrates

Similar to previous work (DeQuach et al., 2011; Baiguera et al., 2014; Sood et al., 2016), we manufactured mixed 9.5 mg/mL BMX/GTX hydrogels by diluting the tissue-derived BMX hydrogel in GTX and cell culture medium. Quantities were calculated so that the volume of GTX would not exceed 50% of the total hydrogel volume, and the remaining 50% was constituted by BMX. For example, to prepare 250µL BMX hydrogel, 125 µL of 15 mg/mL GTX, 100 µL of 5 mg/mL BMX and 25 µL of cell culture medium were combined. ECM hydrogels were plated into cell culture plate wells and incubated overnight at 37.0 °C to achieve gelation.

Cells were diluted in culture medium to a concentration of 10 million cells/mL and bioprinted into the preformed three-dimensional hydrogels, placing roughly 100 cells at each injection site using our custom 3D bio-printing system (Mollica et al., 2019). Cell clusters were bioprinted in a 3 × 10 grid, 300 µm distance between each column and 1 mm between each row. Cells were dispensed in glass needles pulled to a tip diameter of 100 µm with a P-1000 micropipette puller (Sutter Instrument; Novato, CA). Cell culture medium was added to each well after printing cells within the substrate, and samples were incubated at 37.0 °C.

### *In vivo* transplants

All mice were housed in an Association and Accreditation of Laboratory Animal Care-accredited facility per the NIH Guide for the Care and Use of Laboratory Animals. The Old Dominion University Institute Animal Care and Use Committee approved all experimental procedures.

mESC-Olig2-GFP cells were 3D printed in BMX or GTX hydrogels and cultured for 3 days. The endogenous epithelium of the inguinal (#4 and #9) mammary glands of sedated 3-week-old female athymic nude mice was removed as previously described (Bruno et al., 2017), and cells embedded in the three-dimensional matrices were placed into the cleared mammary fat pad. After 4 weeks of incubation, the tissue was removed, fixed, and embedded in paraffin for histological analysis.

For direct *in vivo* injections, BMX was diluted 1:2 in DMEM/F12 mixed with 50,000 hiPSC or hNSCs and injected into the cleared mammary fat pad. After 10 weeks, the tissue was removed, fixed, whole mounted, and embedded in paraffin for histological analysis as previously described (Bruno et al., 2012; Bruno et al., 2014; Bruno et al., 2017).

### Nucleic acid extraction and gene expression analysis

RNA extraction was performed using TRIzol^®^ reagent (Thermo Fisher Scientific) following the manufacturer’s protocol. Genomic DNA was digested using Deoxyribonuclease I, Amplification Grade (Thermo Fisher Scientific), following the manufacturer’s protocol. RNA was then quantified using a NanoDrop 2000 (Thermo Fisher Scientific). RNA was reverse transcribed using the High-Capacity cDNA Reverse Transcription Kit (Thermo Fisher Scientific), per the manufacturer’s protocol.

qRT-PCR was carried out using TaqMan^®^ Gene Expression Assay (Applied Biosystems) on a StepOnePlus™ Real-Time PCR System (Applied Biosystems; Foster City, CA) per manufacturer’s protocol. Human and mouse specific TaqMan primer probe sets (Thermofisher) were used to detect SOX2, Nestin, Doublecortin (DCX), Beta III Tubulin (TUBB3), GALC/OLIG2, and GFAP. All experiments were performed in triplicates, and each sample was sub-sampled three times. Gene expression analysis was conducted using the 2^−ΔΔCt^ of the average Ct for each subsample.

### Immunostaining and imaging

2D cultures were fixed in 10% formalin for 20 min and permeabilized with 0.1% NP40 for 10 min at room temperature. Samples were blocked in 10% goat serum for 60 min. Primary antibodies were diluted in 1% goat serum according to the manufacturer’s instruction, and, following application, they were incubated overnight at 4 °C. Primary antibodies used were: anti-Nestin (10C2, Thermofisher), anti-Sox2 (ab97959, Abcam), anti-Pax3 (38–1801, Thermofisher), anti-MAP2 (MA5-12826, Thermofisher), anti-TUJ1/Beta III Tubulin (ab7751, Abcam), anti-DCX/doublecortin (48–1200, Thermofisher), anti-TH/tyrosine hydroxylase (ab112, Abcam), anti-BDNF (ab108319, Abcam), anti-Synapsin 1/SYN1 (ab645581, Abcam), anti-GFAP (ab7260, Abcam), anti-OLIG2 (ab109186, Abcam), and anti-CD44 (ab6124, Abcam). AlexaFluor™ 488 and 568 conjugated secondary antibodies (Thermo Fisher Scientific) were added, and the samples were incubated in the dark, at room temperature, for 60 min. Nuclei were counter-stained with DAPI.

Three-dimensional cell culture samples were fixed with 10% formalin for 1 hour, then embedded in paraffin and sectioned by Histoserv Inc. (Germantown, MD). One section of each sample was stained with hematoxylin and eosin to define the nuclear content or cellular morphology. Immunostaining of the remaining slides was performed as above with the addition of an antigen retrieval step using citrate buffer for 25 min.

All imaging was performed and processed using a Zeiss Axiocam and Zeiss Zen Software. Image quantitation of 3D prints was performed using Zen Software to measure fluorescent intensity at each print location. Areas lacking cells were used to subtract the background.

### Statistical analysis

All experiments were carried out in at least triplicate. Statistical analysis and graphical data representation were performed with Prism 7 (GraphPad Software; La Jolla, CA). For gene expression data, standard two-tailed T-Tests were used to determine significant differences between GTX and BMX treatments for each gene. A Two-Way ANOVA with Sidak’s Multiple Comparison *post hoc* was performed for fluorescence quantitation of GFP. A Mann-Whitney U test was performed to compare the nuclear to cytoplasm ratio between mESCs grown in GTX vs. BMX. A Fisher’s Exact test was used to determine the significance between take rates *in vivo* inoculations.

## Results

### Brain ECM supports differentiation of stem cells in 2D culture

**FIGURE 1:**
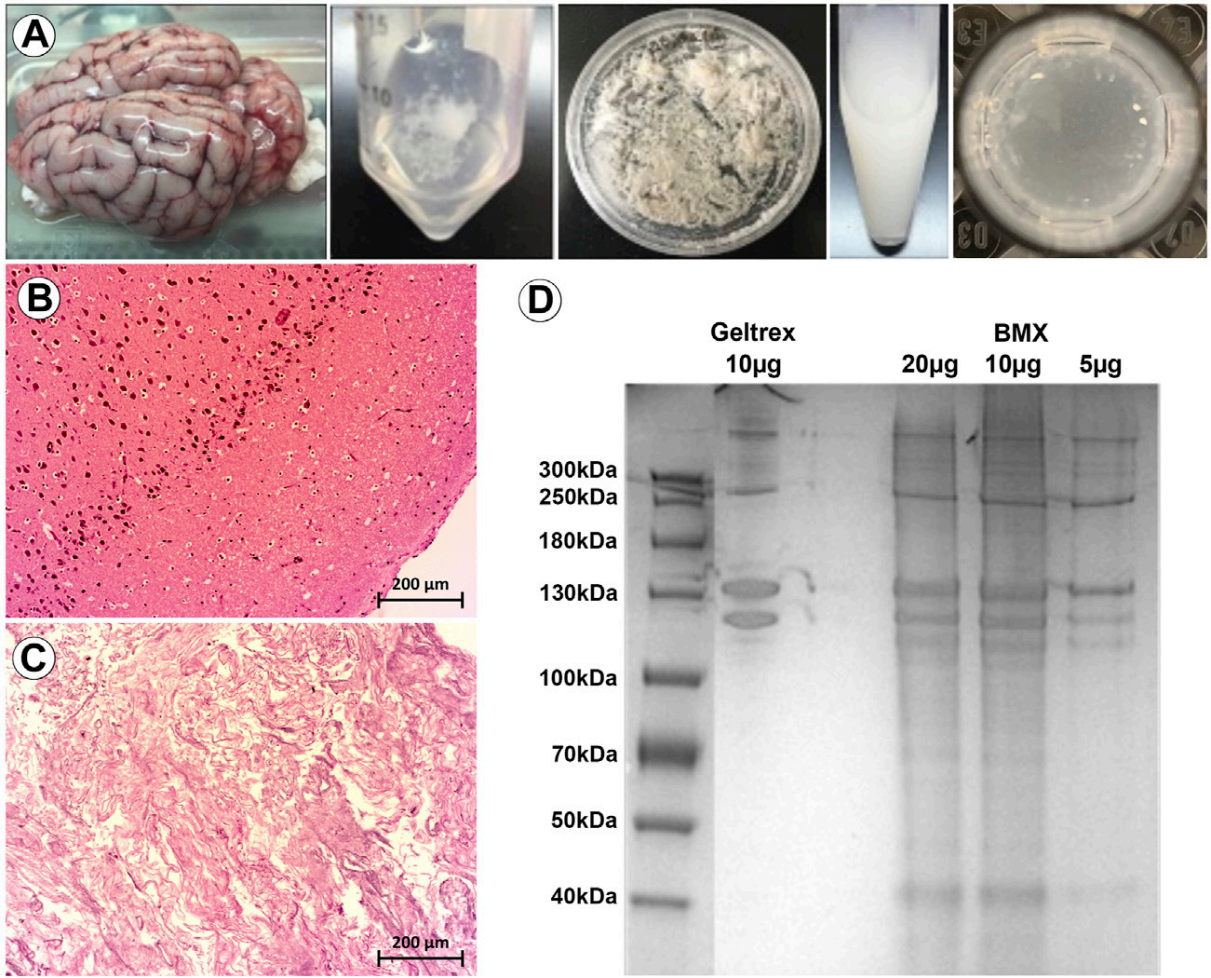
Porcine brain-derived ECM extracts maintain tissue specific proteins and spontaneously gel. **(A)** From left to right; whole brain extracted from porcine head. Brain tissue following decellularization. Brain tissue following lyophilization. Brain tissue following the pepsin digestion process. Hydrogel spontaneously formed after incubation of the final product at 37 °C. **(B)** H&E staining of native brain, with visible cell nuclei. Size bar = 200 µm **(C)** H&E staining of decellularized brain, with cell nuclei not visible. Size bar = 200 µm **(D)** PAGE assay for the analysis of the hydrogel’s protein content compared to GTX.

We first developed a modified version of previously published extraction protocol (DeQuach et al., 2011; Crapo et al., 2012; Medberry et al., 2013; Simsa et al., 2021), which resulted in a self-gelling extract from porcine brain (BMX; Figure 1A). Following decellularization, nucleic acids were significantly reduced (Figures 1B,C). The protein composition was consistent with a basement membrane-rich ECM with a unique complement of smaller proteins (Figure 1D). To confirm our gels were in alignment with previous studies that have extensively characterized the protein content of porcine brain extracts (DeQuach et al., 2011; Medberry et al., 2013; Sood et al., 2016; Mollica et al., 2019), we examined the rheological properties of the resulting gel. The BMX exhibited a G of 2.03 pa ( ± 0.71) and a G″ of 1.38 pa ( ± 0.73), which was too liquid to support printing with our 3D bioprinting system adequately. Because variations in stiffness can have a profound impact on cellular behaviour (Chaudhuri et al., 2020; Ge et al., 2021) we chose to standardize this variable by aligning with previous studies (DeQuach et al., 2011; Crapo et al., 2012; Simsa et al., 2021). Thus, we made a more structurally suitable hydrogel with a 1:1 mixture BMX with growth-factor reduced basement membrane extracts (GTX) which resulted in a G′ of 16.62 ( ± 0.87) and a G″ of 2.22 ( ± 0.27).

**FIGURE 2:**
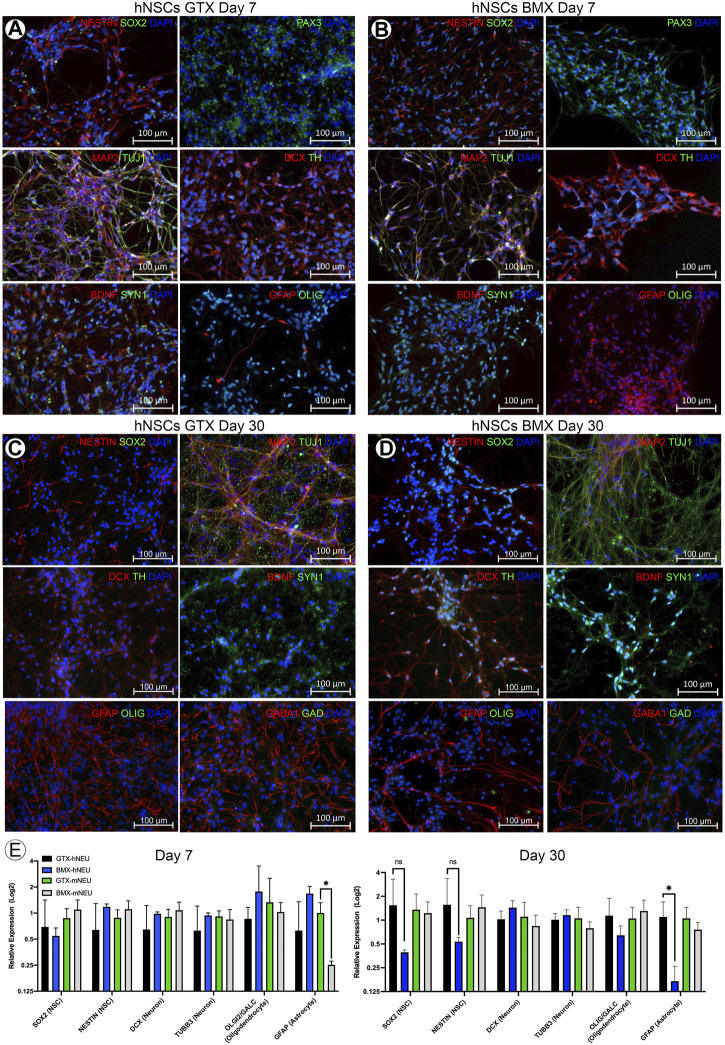
2D coatings of BMX supports neural differentiation of NSCs. **(A,B)** ICC demonstrating expression of neural markers at 7 days neural differentiation in hNSCs on GTX **(A)** and BMX **(B)** coated plates. **(C,D)** ICC demonstrating expression of neural markers at 30 days neural differentiation in hNSCs on GTX **(C)** and BMX **(D)** coated plates. **(E)** qRT-PCR of neural markers after 7 and 30 days in mNSC and hNSCs cultured on GTX or BMX. *p = <0.05.

We next investigated whether BMX alone could facilitate or influence the neural differentiation process in monolayer stem cell cultures. We found that BMX coatings would not support the attachment or long-term survival of mESCs or human iPSCs (not shown). However, we found both human (Figure 2) and mouse (Supplementary Figure S1) NSCs could attach and survive on BMX coated plates. To test the effectiveness of BMX on 2D differentiation of mNSCs and hNSCs, we compared GTX or BMX coated plates on short-term (7 days) and long-term (30 days) neural differentiation (Figure 2, Supplementary Figure S1). We observed increased expression of neural cell differentiation markers at both the protein and mRNA levels in both human and mouse cells grown on GTX and BMX coated plates, consistent with robust neural differentiation. The only significant differences noted between the substrates was in GFAP mRNA expression at 7 days in mouse cells and 30 days in human cells (Figure 2E). In both cases, we measured less GFAP expression in the cells grown on the BMX substrate. There was also a marked, but not significant, reduction in the expression of NSC markers SOX2 and Nestin in human NSCs grown on BMX compared to GTX at 30 days, likely due to high variability in the expression of these markers in cells grown on GTX. The reason or significance of the difference in GFAP expression is not apparent, though it is likely due to temporal differences in the differentiation process. The lack of an observable difference in protein expression of GFAP protein at 30 days (Figures 2C,D) supports this interpretation. These findings demonstrate that BMX is equally effective as GTX in supporting the differentiation of human and mouse NSCs when used as a thin coat in 2D with the potential to be differentially driving cells toward various neural lineages.

### 3D bioprinting ESCs in BMX containing hydrogels promotes differentiation towards the neural lineage

**FIGURE 3:**
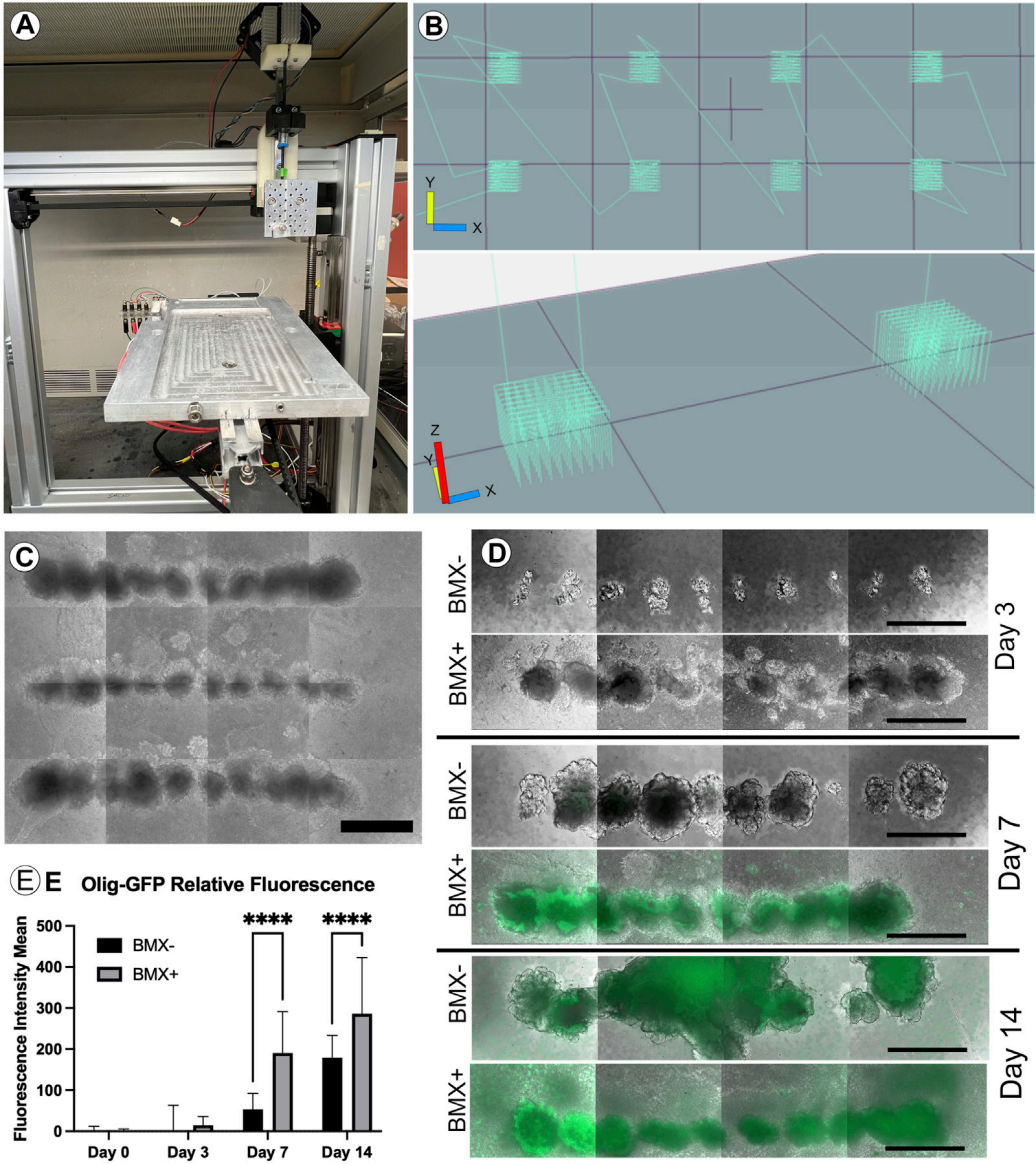
Addition of BMX to hydrogels promotes neural differentiation of 3D bioprinted mESC-OLIG2-GFP cells. **(A)** Image of the low cost, adaptable 3D bioprinter used in these studies. **(B)** Graphical representation of GCODE routine used to print 3D arrays in 24-well dishes. **(C)** Representative image of full-size array of mESC-OLIG2-GFP printed into BMX + hydrogels in a 24 well dish. Scale bar = 500 µm **(D)** Growth and development of 3D cellular structures from mESC-OLIG2-GFP 3D bioprinted arrays over 14 days in BMX+ and BMX-hydrogels. **(E)** Relative fluorescence over 14 days of 3D bioprinted arrays of mESC-OLIG2-GFP printed in BMX+ and BMX-. ****p=<0.0001 Scale bar = 500 µm.

ECM and its normal 3D configuration can profoundly affect cellular differentiation (Bruno et al., 2017). To examine this, we first needed to adapt our novel 3D culture system to evaluate the effect of BMX on 3D cultures. Traditional 3D cell culture involves either the manual injection of a cell solution within a three-dimensional substrate or the mixture of cell solution with the substrate prior to gelling. Either method produces random cell clusters that are difficult to control from experiment-to-experiment and from a cell-to-cell interaction standpoint. Our 3D bioprinter system allows for the precise placement of cells within any 3D hydrogel with minimal cellular shear stress resulting in negligible effects on cell viability and cellular differentiation (Reid et al., 2016). The printer’s precise placement of cells can facilitate a high-throughput array of repetitive 3D structures (Figures 3A–C). To test the effect of BMX on differentiation, we compared our 1:1 BMX:GTX hydrogels (BMX+) to standard GTX-only hydrogels (BMX-). We printed mESCs engineered to express GFP only when the OLIG2 promoter was activated (mESC-OLIG2GFP; Field (Xian et al., 2003)) into BMX+ and BMX-hydrogels to monitor differentiation. As mentioned previously, while OLIG2 is traditionally a maker of oligodendrocytes, the reporter can be used as a general marker of neural differentiation (Mizuguchi et al., 2001). Thus, this novel system allowed us to test the impact of BMX on pluripotent stem cell differentiation to neural lineages in real-time.

The cells were printed and cultured in mESC + LIF growth medium and could survive and proliferate in both substrates. Cell growth was followed throughout the 14 days by monitoring the expression of the Olig2-GFP marker by live cell fluorescence microscopy. As expected, no to minimal GFP expression was visible at day 0 or 3, but a marked increase occurred in cells grown in BMX + hydrogels by day 7 and was detectable in both substrates by day 14 (Figures 3D,E). Notably, GFP expression was significantly higher in mESC-OLIG2-GFP cells grown in BMX + hydrogels on both day 7 and day 14 compared to the same cells grown in BMX-hydrogels, consistent with the hypothesis that BMX directs differentiation of pluripotent stem cells preferentially towards neural cell fates.

**FIGURE 4:**
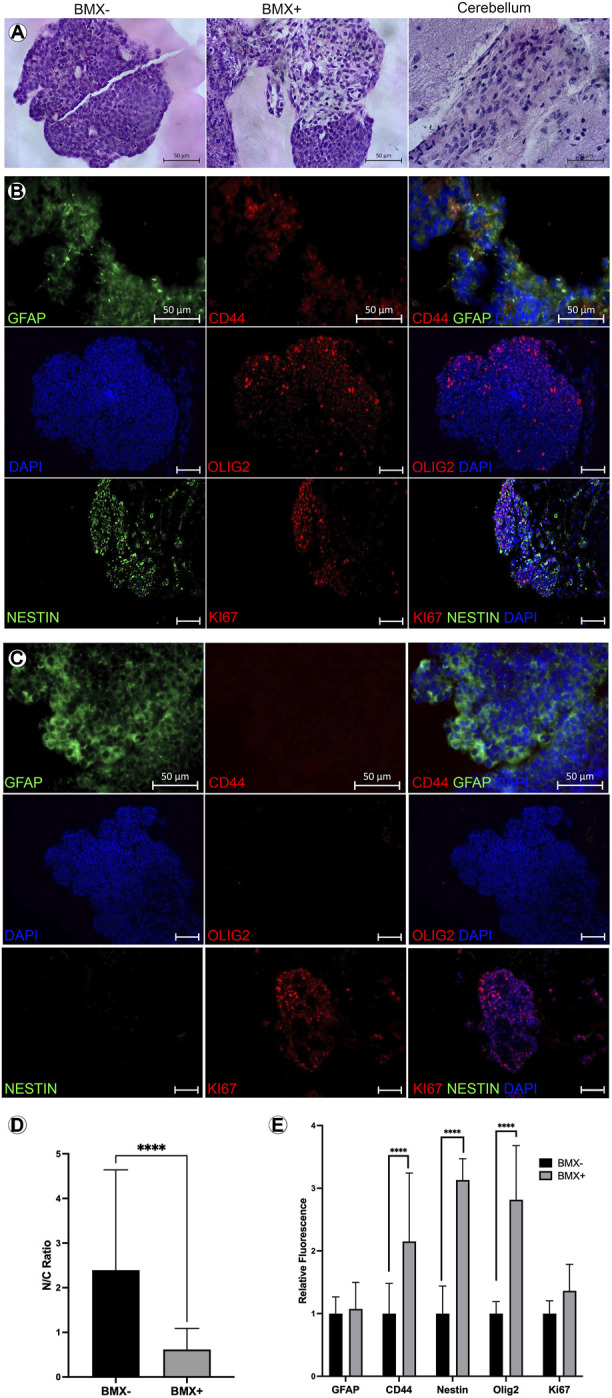
Addition of BMX to hydrogels alters morphological and induces neural differentiation markers in 3D bioprinted mESCs. **(A)** H&E images of mESC 3D bioprinted 3D cellular structures in BMX- (left) BMX+ (center) hydrogels compared to H&E stain of a section of cerebellum (right). Scale bar = 50 µm. **(B)** IHC of mESC neural structures from BMX-hydrogels expressing GFAP, CD44, OLIG2, Nestin, and Ki67 with overlay DAPI nuclear stain. Scale bar = 50 µm **(C)** IHC of mESC neural structures from BMX + hydrogels expressing GFAP, CD44, OLIG2, Nestin, and Ki67 bwith overlay DAPI nuclear stain. Scale bar = 50 µm. **(D)** Quantificaiton of cytoplasmic to nuclear ratio compared between mESC neural structures 3D bioprinted in BMX- (black bar) or in BMX+ (grey bar) hydrogels ****p=<0.0001 **(E)** Quantification of GFAP and CD44 expression from sections of BMX+ and BMX-hydrogels mESC 3D cellular structures *p=<0.05.

To further explore the differentiation and morphology of the cells in 3D, we evaluated histological sections of the 3D cultures 14 days post-print. We noted an altered morphology of mESC-OLIG2-GFP colonies grown in the BMX + versus BMX-hydrogels (Figure 4A). Specifically, all mESC-OLIG2-GFP colonies developed within the BMX-substrate presented tightly packed cells with a high nucleus-cytoplasm (N:C) ratio, while mESC grown in BMX + hydrogels formed colonies with statistically significant reduced cellular density and N:C ratio (Figures 4A, D). This result is consistent with a higher degree of cellular differentiation in the presence of BMX. Furthermore, the cellular morphology of mESCs grown in BMX suggests differentiation towards neuroglial cells, as the morphology is comparable to native brain cerebellum (Figure 4A). Immunological staining found increased expression of the NSC marker nestin, astrocyte marker CD44, and confirmed increased expression of OLIG2 in cells grown in BMX + as compared to BMX-hydrogels (Figures 4B, C, E). No significant difference in GFAP expression or the proliferation marker ki67 was seen between the substrates, although both were present in samples from each substrate. These findings, combined with the higher activation of OLIG2-GFP (Figures 3D, E) and the observation of altered cellular morphology, suggest that the presence of the brain-derived substrate directs mESC differentiation towards neural lineages. The continued expression of ki67 and nestin suggest the majority of the cells remained in primitive neural states. However, the rapid conversion from pluripotency to strong OLIG2 expression and almost uniform nestin expression clearly indicate the BMX’s potent influence on the differentiation of mESCs.

Conversely, we saw no significant impact of BMX on the differentiation of mNSCs (Supplementary Figure S2) in 3D. We interpreted this to mean that BMX had an insignificant effect because NSCs are already primed for neural differentiation, and the medium supporting the differentiation directs non-specific neural differentiation. This is consistent with the interpretation that BMX favours non-specific neural differentiation from pluripotent state. Overall, these data describe a novel system for studying neural differentiation in 3D and support the hypothesis that tissue-specific ECMs can direct differentiation of stem cells to cell types found within the original tissue.

### BMX supports neural differentiation *in vivo*

**FIGURE 5:**
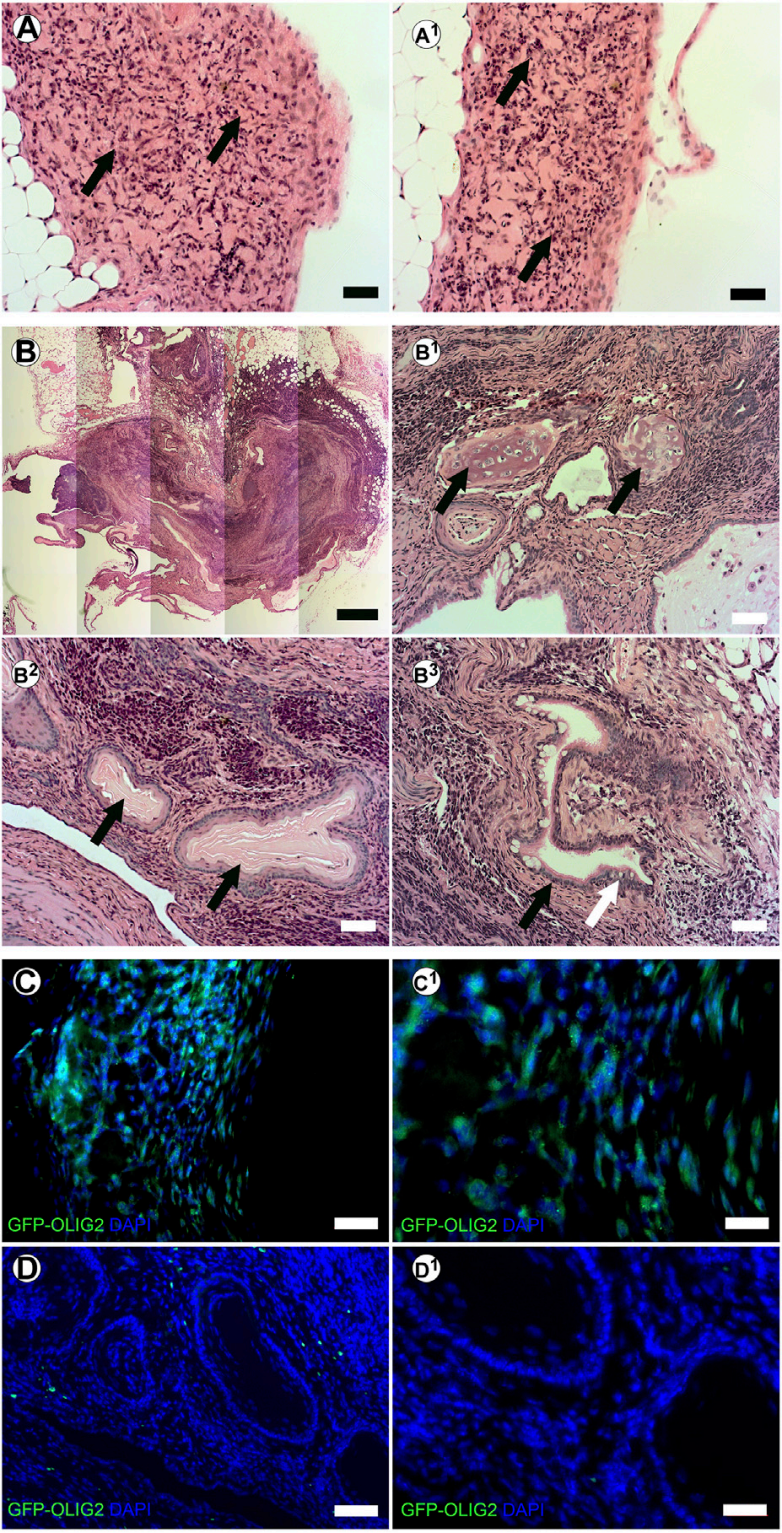
In vivo transplanted 3D bioprinted arrays in BMX-containing hydrogels form consistent neural growths. (A-A1) H&E staining of neural structures resulting from transplanted 3D bioprinted mESC in BMX+. Scale bar = 50 µm **(B)** H&E staining of disorganized teratoma from transplanted 3D bioprinted mESCs in BMX- (B1) Cartilage in Teratoma (arrows) (B2) Ectodermal squamous epithelium and cuboidal basal cell structures (arrows) (B3) Endodermal columnar epithelial (Black arrow) and goblet cells (White Arrow). Scale bar = 50 µm. **(C)** Immunofluorscence staining for GFP representing OLIG2-GFP expression in transplanted mESC neural structures in BMX + hydrogels. Scale bar = 50 µm (left) 20 µm (right). **(D)** Immunofluorescence staining for GFP representing GFP-OLIG2 expression of transplanted mESCs in BMX-hydrogels. Scale bar = 50 µm (D1) 20 µm.

We have previously shown that tissue-specific ECM can direct stem cell differentiation within the context of the cleared mammary fat pad *in vivo* (Bruno et al., 2017). Therefore, we evaluated the potential of creating stem cell derived neural structures *in vitro* and transplanting them *in vivo* using our system. mESC-OLIG2-GFP were bioprinted into BMX + or BMX-hydrogels and kept in culture for 3 days. Bioprinted structures were cut from hydrogels and surgically implanted into cleared mammary fat pads of 3-week-old nu/nu mice. After 28 days, no evidence of external growth or GFP expression was seen in mammary fat pads that received direct injections of mESC-OLIG2-GFP cells (0/3; not shown). In contrast, 4/6 mammary glands transplanted with bioprinted mESC-OLIG2-GFP structures across both hydrogels had detectable exogenous growths (Figures 5A, B). 2/3 glands transplanted with cells bioprinted into BMX + hydrogels had GFP + detectable outgrowths consistent with neural differentiation (Figures 5A,C). Two outgrowths resulted from mESC-OLIG2-GFP in BMX-hydrogels, one formed a teratoma with characteristic differentiation down all three germ layers (Figure 5B-B^3^), and both largely lacked GFP expression. It should be noted that at 3 days in culture, no GFP was detectable under these conditions (Figure 3C), and thus, this activation of GFP in the BMX + group occurred *in vivo*. These findings demonstrate the utility of transplanting 3D bioprinted cellular structures *in vivo* and support the conclusion that BMX directs mESCs to differentiate down a primarily neural/glial cell path *in vivo*.

**FIGURE 6:**
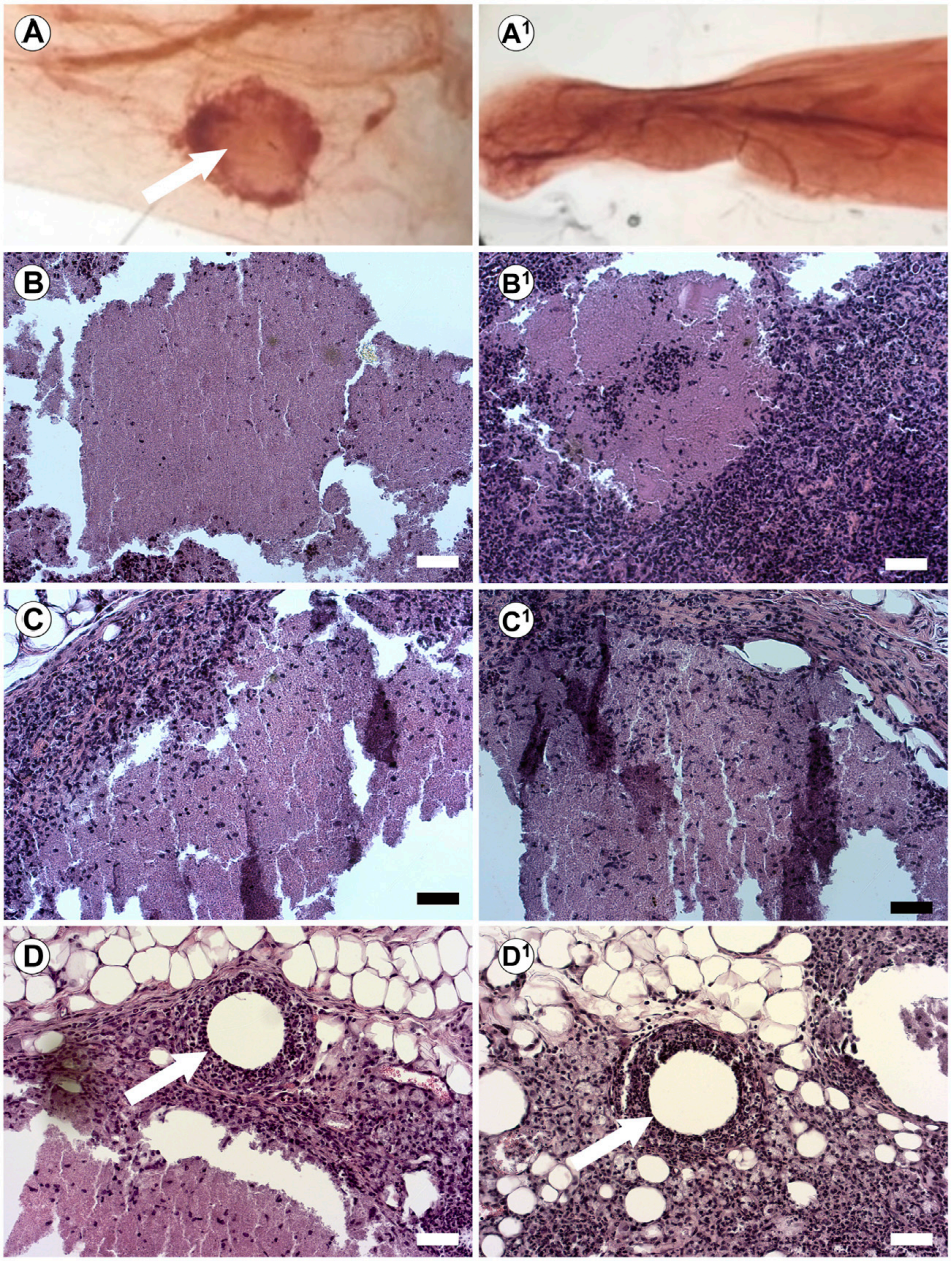
*In vivo* Injections of hiPSCs and hNSCs with BMX form consistent neural structures. **(A)** Whole mount of mouse mammary fat pad 4 weeks post injection of hiPSC outgrowth with BMX (A; Arrow) or mammary ECM (A1). **(B,C)** H&E stain of growths from injected BMX with hiPSC (B-B1) or hNSCs (C-C1) demonstrating neural differentiation into immature brain tissue. Scale bar = 50 µm. **(D)** Neural rosettes in NSC injection with BMX (arrow) (D1) Neural rosettes in iPSC injected with BMX (arrow). Scale bar = 50 µm.

Our previous studies in the breast used injectable ECM to direct differentiation *in vivo* without prior bioprinting. Furthermore, we wanted to test the influence of our substrate on human pluripotent cells. We have previously demonstrated that injection of hiPSC into the cleared mammary fat pad with Geltrex results in teratomas (Mollica et al., 2018b). Therefore, here we tested the capacity of the BMX to direct differentiation of human induced pluripotent stem cells (hiPSCs) and human iPSC-derived NSCs (hNSCs) *in vivo*. We mixed cells with unsolidified BMX or mammary ECM (Bruno et al., 2017) and injected them into the cleared mammary fat pad of 3-week-old nu/nu mice. After 10 weeks, glands were removed and examined for exogenous outgrowths. When inoculated with mammary ECM, no outgrowths developed with hiPSC (0/5; 0/5) and hNSC (0/5; 0/5). However, when inoculated with BMX, 6/8 iPSC injections and 6/8 NSC injections formed outgrowths (Table 1; Figure 6; *p* = 0.021). Histological analysis of the iPSC and NSC derived outgrowths demonstrated that both had nearly identical morphologies consisting of uniform neural differentiation with areas of primitive brain tissue (Figures 6B,C) and neural rosettes (Figure 6D-D^1^). Notably, there was no evidence of teratoma or multilineage differentiation with the outgrowths derived from the hiPSCs.

**TABLE 1:** Take rates from *in vivo* dispersed cell inoculations. *p = 0.021.

Cells	ECM	Outgrowths/Inoculations
hNSC	Mammary	0/5
hNSC	BMX	6/8*
hiPSC	Mammary	0/5
hiPSC	BMX	6/8*

These results are the first demonstration that BMX without additional differentiation stimulates, directs differentiation of pluripotent stem cells to neural cell fates *in vivo.* Furthermore, the success of these transplants underscores the benefits of our combined bioprinting/ECM system allowing for analysis of the effects of ECM on cellular differentiation both *in vitro* and *in vivo*.

## Discussion

To fully understand the role of ECM in cell fate determination, better model systems are needed. To this end, our group has worked extensively to develop 3D culture techniques using our bioprinting platform and tissue-specific ECMs (Reid et al., 2016; Sachs et al., 2017b; Reid et al., 2018; Bruno et al., 2019; Mollica et al., 2019; Reid et al., 2019). The printer is open source and assembled by modifying inexpensive commercially available 3D fused deposition method printers using 3D printed parts. Files to produce the printer are on our website (odustemcell.org), making the system easily accessible to all laboratories. The significant advantage of our system over traditional 3D culture is that it allows for accurate placement of cells—ranging from a single cell to 100s of cells per print site—within 3D matrices rather than relying on random cellular interactions. Furthermore, we have established that the pulled glass design limits shear stress on cells virtually eliminating cell damage and differentiation (Reid et al., 2016). The present work provides a proof of principle approach to our model system for studying stem cell/brain ECM interactions. Differentiation can be monitored in real-time using reporter cells such as the mESC OLIG2 used in these studies. Furthermore, because of the nature of the printing process, high throughput screens can be performed in a single gel, reducing the waste of difficult-to-obtain tissue-specific ECM.

There would be a broad scientific utility from readily transplantable micro-tissues or organoids for the study of both disease and normal tissue function (Ozbolat, 2015; Sun et al., 2023). Here, we establish a framework for *in vivo* transplantation of bioprinted 3D structures where our prints were easily dissected and transplanted into a mouse. Conversely, before gelation, we also demonstrated that cells can be mixed with the ECM directly and injected. To the best of our knowledge, this is the first use of the mammary gland for *in vivo* transplant of bioprinted 3D cellular structures of any origin. The support of neural tissue in the mammary gland is also novel. We chose to transplant into the cleared mammary fat pad of mice because of our previous experience in tissue-specific injections into the gland, and we hypothesized that the supportive nature of the fat pad (supportive stromal component, blood supply, etc.) would be an ideal location to support structures/organoids even of non-mammary origin. Furthermore, the ease of accessibility to the mammary gland and the simplicity of the surgery makes it an attractive alternative to the kidney capsule.

Using this system, we demonstrate that brain ECM can influence the differentiation of pluripotent stem cells. We saw this first-of-its-kind observation in 3D cultures and *in vivo* transplants. In addition to increased OLIG2 expression, BMX also induced expression of CD44 and nestin, the latter was almost uniformly expressed throughout the structures at 14 days. The lack of morphological or immunological evidence for neuronal differentiation and the expression of ki67 supports a general differentiation towards primitive neural lineages. Interestingly, in 2D cultures, less expression of the astrocyte marker OLIG2 was seen, seemingly counter to the 3D experiments. However, this finding may be an artifact of 2D culture that fails to mimic the microenvironment sufficiently or may represent fluctuations in the temporal expression of OLIG2 mRNA, which have been identified in NSCs and oligodendrocytes (Kageyama et al., 2019). Importantly, we observed no differences in protein expression. We were unable to explore the role of BMX in pluripotent cellular differentiation in 2D because BMX coating would not support attachment by mESCs or hiPSCs even when mixed in 1:1 mixtures with GTX (data not shown). This is likely due to the much lower concentration of pro-survival signaling within the BMX substrate (Simsa et al., 2021). Interestingly, we found that for the pig brain ECM isolation, batching was not necessary to maintain consistent biological activity. This also seemed to be the case for previous reports as they also did not note batching as a requirement (Crapo et al., 2012; Medberry et al., 2013; Simsa et al., 2021). Furthermore, while we chose to standardize the overall stiffness of our gels, this critical variable could facilitate future tunability that would enable altering/enhancing neural cell fates further. Also, employing variations in extracts from different brain regions alongside alterations in the stiffness could hypothetically tune the ECM hydrogels to promote formation of brain specific region differentiations.

*In vivo*, the BMX directed specific neural differentiation in both iPSCs and NSCs. The *in vivo* findings are consistent with previous findings that ECM from the mammary gland could prevent teratoma formation and direct differentiation of pluripotent cells to a mammary cell fate (Bruno et al., 2017). These findings are the first of their kind and underscore the importance of ECM in fate determination. The transplants were maintained for 4 weeks for the bioprinted structures and 10 weeks for the direct injections. These times were based on assumptions of the time it would take for structures to form and/or die based on our experience with the mouse mammary fat-pad model and our approved animal protocols. In all cases, there was no sign of necrosis despite the size and density of the structures demonstrating good nutrient and blood flow. Therefore, it appears the structures could survive indefinitely *in vivo*. Unfortunately, our process of whole mounting the glands to identify structures within the fatty tissue combined with the density of the structures impeded our ability to do in-depth immunohistochemical characterization of the outgrowths. However, the morphological evidence was clear, demonstrating primitive neuronal structures, including neural rosettes and neural epithelium. Furthermore, the fact that 75% of BMX-containing transplants of either hiPSCs or hNSCs formed these neural structures while none in the control groups survived underscores the biological effect of BMX. Future studies will explore if long-term engraftment leads to alterations in differentiation as well as the capacity of the outgrowths to form secondary growths if excised and transplanted into a new fat-pad. Such studies will also help facilitate more thorough molecular analysis of cellular differentiation through technologies such as single-cell sequencing.

The system we have developed here could be adapted to identify critical components of the ECM to tease out mechanisms for better control of stem cell differentiation. For instance, specific factors can be added back to a simple basement membrane, such as growth factor reduced GTX, to test individual components of the ECM. Conversely, inhibitory antibodies can be added to prevent interaction with specific molecules. Furthermore, single-cell prints can evaluate molecular changes directly initiated by the ECM. These experiments would eliminate contributing factors from surrounding cells and thus paint a specific picture of the role of ECM-related signaling. Dilution of the BMX into growth media could also be explored both in 3D and in 2D to assess the role of soluble factors within the BMX.

Overall, we have described a novel system combining ECM isolated from the brain with precise cell placement mediated by a simple, low-cost 3D bioprinter. This system can measure differentiation in real time when combined with reporter cells. Furthermore, we developed a novel system for *in vivo* transplantation of bioprinted 3D cellular structures. Using this novel approach, we demonstrate that brain ECM can direct pluripotent cells to differentiate into neural cell lineages. Thus, we have described a novel robust 3D cell culture system to measure stem cell/ECM interactions. Using the system, we demonstrate that brain ECM can impact the differentiation of pluripotent stem cells.

## Data Availability

The authors acknowledge that the data presented in this study must be deposited and made publicly available in an acceptable repository, prior to publication. Frontiers cannot accept a manuscript that does not adhere to our open data policies.
