# Supplementary figures and images for: Combined 3D bioprinting and tissue-specific ECM system reveals the influence of brain matrix on stem cell differentiation
Source: Front Cell Dev Biol. 2023 Oct 20;11:1258993. doi: 10.3389/fcell.2023.1258993 (PMC10623327; doi:10.3389/fcell.2023.1258993)

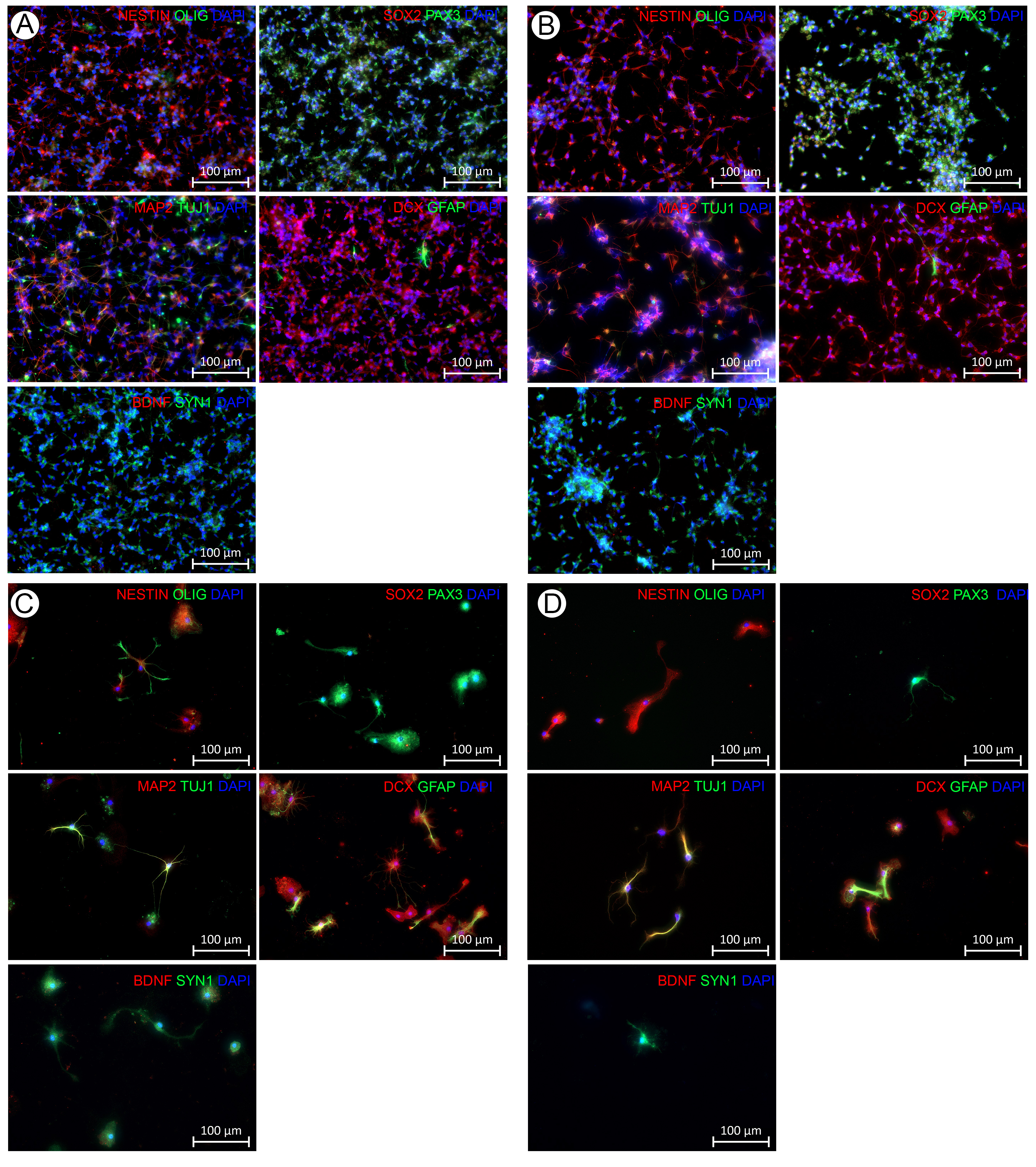

Supplement: Supplementary file 1 [file Image1.jpeg]

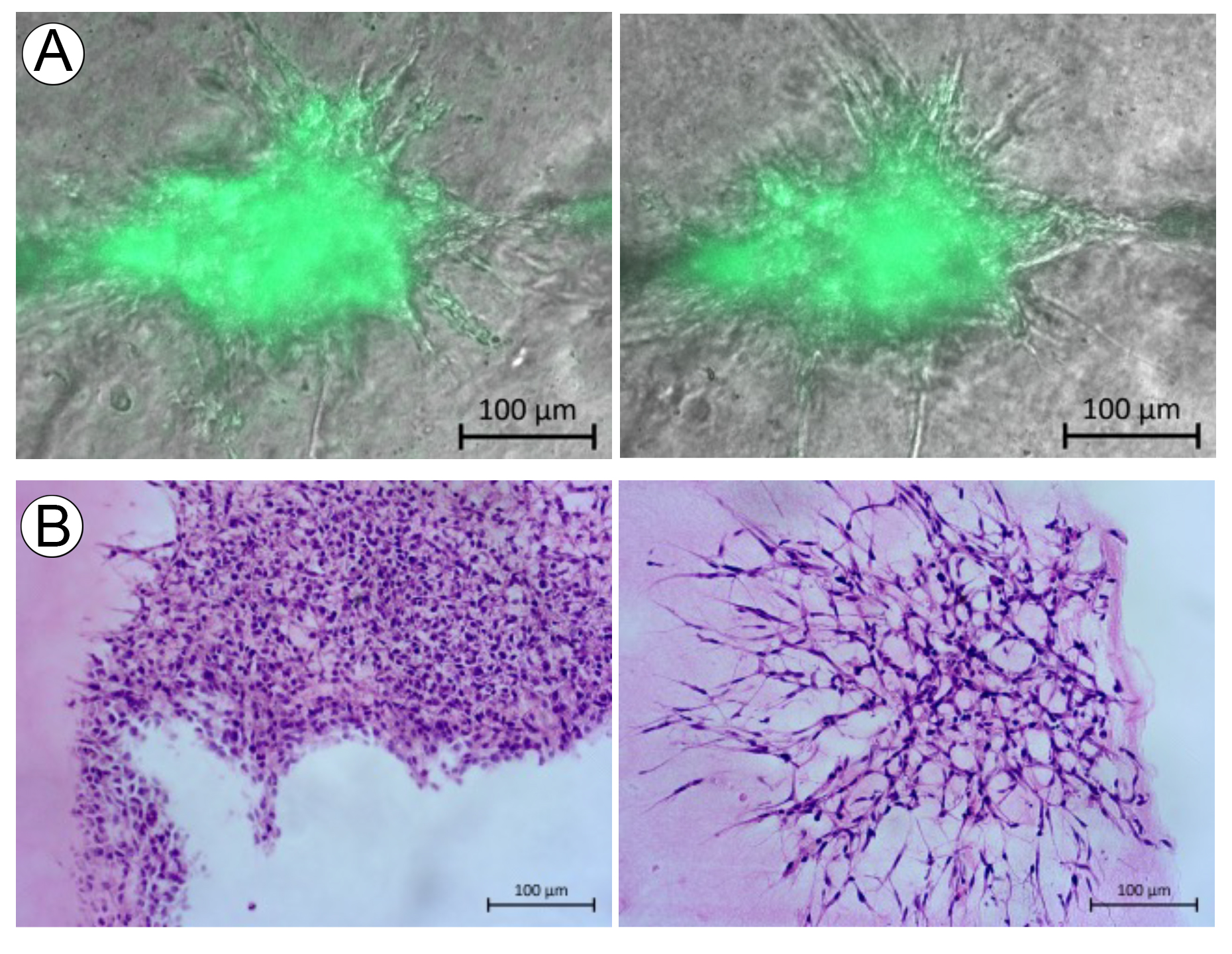

Supplement: Supplementary file 2 [file Image2.jpeg]
